# A New Criterion to Evaluate Water Vapor Interference in Protein Secondary Structural Analysis by FTIR Spectroscopy

**DOI:** 10.3390/ijms150610018

**Published:** 2014-06-04

**Authors:** Ye Zou, Gang Ma

**Affiliations:** Key Laboratory of Medicinal Chemistry and Molecular Diagnosis of Ministry of Education, College of Chemistry and Environmental Science, Hebei University, Baoding 071002, China; E-Mail: yezou.zh@gmail.com

**Keywords:** FTIR spectroscopy, vibrational spectroscopy, protein secondary structure, second derivative, Fourier self-deconvolution

## Abstract

Second derivative and Fourier self-deconvolution (FSD) are two commonly used techniques to resolve the overlapped component peaks from the often featureless amide I band in Fourier transform infrared (FTIR) curve-fitting approach for protein secondary structural analysis. Yet, the reliability of these two techniques is greatly affected by the omnipresent water vapor in the atmosphere. Several criteria are currently in use as quality controls to ensure the protein absorption spectrum is negligibly affected by water vapor interference. In this study, through a second derivative study of liquid water, we first argue that the previously established criteria cannot guarantee a reliable evaluation of water vapor interference due to a phenomenon that we refer to as sample’s absorbance-dependent water vapor interference. Then, through a comparative study of protein and liquid water, we show that a protein absorption spectrum can still be significantly affected by water vapor interference even though it satisfies the established criteria. At last, we propose to use the comparison between the second derivative spectra of protein and liquid water as a new criterion to better evaluate water vapor interference for more reliable second derivative and FSD treatments on the protein amide I band.

## 1. Introduction

Curve-fitting of the Fourier transform infrared (FTIR) spectrum of a protein in the 1700–1600 cm^−1^ amide I region is widely used in the quantitative analysis of protein secondary structures [1,2,3,4,5,6,7,8,9,10,11,12,13,14,15]. When implementing the FTIR curve-fitting approach, resolution-enhancement treatments with second derivative and Fourier self-deconvolution (FSD) are commonly used to resolve the overlapped component peaks corresponding to different secondary structures from the often featureless amide I band [1,2,3,4,5,6,7,8,9,10,11,12,13,14,15,16,17,18]. These two techniques offer two important fitting parameters, peak number and peak frequency. As the protein FTIR spectrum is usually taken under atmospheric conditions, the omnipresent water vapor in the atmosphere can significantly affect the outcomes of the second derivative and FSD treatments. Residual water vapor absorptions in the amide I region can be easily resolved by second derivative and FSD due to the intrinsic narrow bandwidth pertaining to the vibration-rotation modes of gaseous water [3,4,9,17,19,20,21,22]. If this happened, the resolved water vapor peaks could be falsely taken as the component peaks due to protein secondary structures. This will lead to errors in subsequent curve fitting. Therefore, successful elimination of water vapor interference from protein amide I band is an indispensible step to ensure reliable quantitative analysis of protein secondary structures.

The elimination of water vapor interference from protein absorption spectrum can be done in several ways. These methods include purging with dry air or nitrogen during spectral acquisition, subtraction of reference water vapor absorption spectrum from protein absorption spectrum, using sample shuttle to achieve complete atmospheric compensation during spectral acquisition, and using the combined approach of purging and spectral subtraction. Regardless of the actual method that one would choose to eliminate water vapor interference, the success of such elimination must be carefully evaluated by some trusted criteria. For this purpose, when the pioneers had developed the FTIR curve-fitting approach, they had also developed several criteria as quality-controls to ensure that the protein spectrum in the amide I region is negligibly affected by water vapor interference [3,7,20,21,23]. We summarized these established criteria by categorizing them into two types. We refer to the first criterion as the “single-point” criterion [3,7]. With this criterion, the successful elimination of water vapor interference is judged by the disappearance of some characteristic absorption peaks of water vapor either from the original absorption spectrum or from the second derivative spectrum. We refer to the second criterion as the “window-region” criterion [7,20,21,23]. With this criterion, the successful elimination of water vapor interference is judged by a featureless baseline in either the original absorption spectrum or the second derivative spectrum in the protein-absorption-free region between 1850 and 1720 cm^−1^. In practice, these criteria are implemented through the practitioner’s visual inspection. Protein absorption spectrum satisfying these criteria will be considered as being negligibly affected by water vapor interference and ready for second derivative and FSD treatments. The above mentioned criteria are widely used in protein secondary structural analysis by FTIR spectroscopy in the past three decades. However, in this study, we demonstrate that these widely adopted criteria in fact cannot guarantee the reliable evaluation of water vapor interference and a protein spectrum satisfying these established criteria can still be significantly affected by water vapor interference. We provide mathematical reasoning to argue why these established criteria are not reliable and introduce a concept called, “sample’s absorbance-dependent water vapor interference” for our reasoning. We suggest a new criterion that we refer to as a “whole-spectrum” criterion to better evaluate the extent of water vapor interference in the FTIR spectrum to ensure more reliable second derivative or FSD treatment on the protein amide I band during curve-fitting analysis.

## 2. Results and Discussion

**Figure 1:**
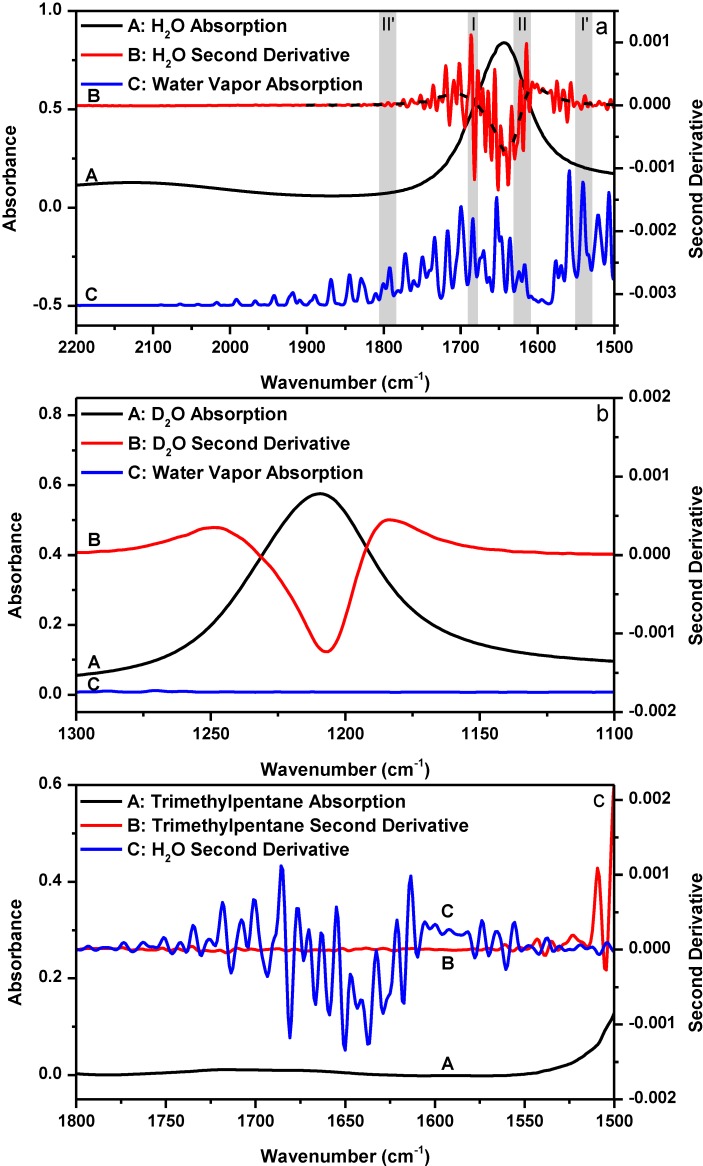
(**a**) FTIR absorption (A: black) and second derivative (B: red) spectra of liquid H_2_O and the absorption spectrum of water vapor (C: blue). Dashed line: theoretical second derivative spectrum of liquid H_2_O; (**b**) FTIR absorption (A: black) and second derivative (B: red) spectra of liquid D_2_O and the absorption spectrum of water vapor (C: blue); (**c**) FTIR absorption (A: black) and second derivative (B: red) spectra of trimethylpentane and the second derivative spectrum of liquid H_2_O (C: blue). Second derivative spectrum is displayed with its resolved peaks pointing downwards. The absorbance of water vapor is in arbitrary unit. Spectral resolution: 4 cm^−1^.

We first argue why the previously established criteria for the successful elimination of water vapor interference is not reliable using a simple example, liquid H_2_O. The bending mode of liquid H_2_O is located in the amide I region around 1645 cm^−1^. From a spectroscopic viewpoint, we can consider liquid H_2_O as some protein mimic which has only one secondary structure. Figure 1a shows the FTIR absorption spectrum of liquid H_2_O bending mode and its corresponding second derivative as well as the absorption spectrum of water vapor in the 2200–1500 cm^−1^ spectral region. Figure 1b shows the FTIR absorption spectrum of liquid D_2_O bending mode and its corresponding second derivative as well as the absorption spectrum of water vapor in the 1300–1100 cm^−1^ spectral region. The comparison between the two figures offers a nice illustration of how water vapor interference affects the outcome of second derivative treatment. As shown in Figure 1b, the second derivative spectrum of liquid D_2_O only gives one resolved peak located at 1207 cm^−1^. By contrast, the second derivative spectrum of liquid H_2_O in Figure 1a appears to contain many resolved component peaks in the 1800–1500 cm^−1^ bending mode region. Since the only difference between the two cases is that there are water vapor absorptions in the H_2_O bending mode region and no water vapor absorption in the D_2_O bending mode region, it is obvious that the resolved peaks in the second derivative spectrum of H_2_O in Figure 1a are the artifacts due to water vapor interference. The dashed line passing through the original second derivative spectrum of liquid H_2_O in Figure 1a shows in principle what the second derivative spectrum of liquid H_2_O should have looked like if there was no water vapor interference in the H_2_O bending mode region. This dashed line was obtained by first over-smoothing the absorption spectrum of liquid H_2_O for three times using a 25-point window and then performing the second derivative treatment using a 25-point window. Obviously, without knowing the significant impact of water vapor interference on the outcome of second derivative treatment, one may falsely take the resolved artifact peaks in Figure 1a as some true component peaks.

**Figure 2:**
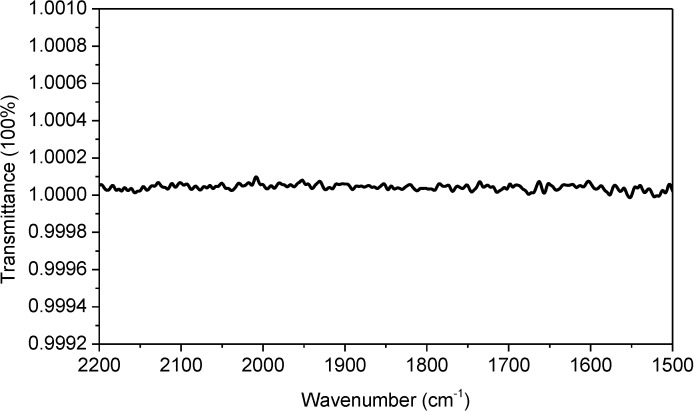
100% transmittance spectrum. This spectrum was taken with an empty optical path and with closed sample compartment. The optical bench of our FTIR spectrometer is a sealed design. This spectrum can be repeatedly obtained with ease under our lab conditions. Spectral resolution: 4 cm^−1^.

The absorption spectrum of liquid H_2_O in Figure 1a was taken with an FTIR spectrometer equipped with a sample shuttle. Using the sample shuttle is mathematically equivalent to conducting reference subtraction with a subtraction factor of 1. As demonstrated in Figure 2, the sample shuttle can ensure a constant water vapor concentration during spectral acquisition, thus resulting in complete atmospheric compensation between sample scanning and reference scanning. Therefore, one may wonder why complete atmospheric compensation is not observed in Figure 1a and why the absorption spectrum of liquid H_2_O is still terribly affected by water vapor interference. In the following, we provide a qualitative interpretation as well as a mathematical reasoning for this surprising observation.

In Figure 1a, we shade and mark two pairs of spectral regions (*i.e.*, region I *versus* region I', and region II *versus* region II'). In each pair, the absorbance of water vapor in the two regions are similar, but the absorbance of liquid H_2_O in the two regions are quite different. In principle, the two second derivative spectra of liquid H_2_O in the paired spectral regions should be affected by water vapor interference to a similar extent upon atmospheric perturbation as the absorbance of water vapor in the two regions are similar. However, we can easily tell from the four marked regions that this is not the case by using the “oscillating” magnitude of the original second derivative signal relative to the reference (*i.e.*, the dash line in Figure 1a) as a qualitative indicator for the extent of water vapor interference. In Figure 1a, the second derivative signal magnitude in region I is more intense than that in region I'; and the second derivative signal magnitude looks obvious in region II but negligible in region II'. Considering the differences among these regions with respect to the absorbance of liquid H_2_O, we can deduce that the extent of water vapor interference at each frequency must highly depend on the absorbance of liquid H_2_O at that frequency; and larger absorbance of liquid H_2_O apparently results in more pronounced water vapor interference in the second derivative spectrum. We here introduce a concept that we refer to as “sample’s absorbance-dependent water vapor interference” to describe the above observation. We should keep in mind that sample’s absorbance means liquid H_2_O’s absorbance, not water vapor’s absorbance. We can further illustrate our discovery in an alternative way by performing the following experiment. Since the sample’s absorbance can abnormally magnify water vapor interference in second derivative spectrum, if we take the FTIR spectrum of some sample which has no absorption in a spectral region, we should see the disappearance of the sample’s absorbance-dependent water vapor interference phenomenon in the second derivative spectrum in that spectral region. Figure 1c shows the absorption and second derivative spectra of 2,2,4-trimethylpentane, a hydrocarbon compound which basically has no absorption in the 1800–1550 cm^−1^ spectral region. The second derivative spectrum of liquid H_2_O was re-shown here for comparison at the same scale. The absorption spectrum of 2,2,4-trimethylpentane was taken under the same condition as liquid H_2_O, *i.e.*, using sample shuttle to ensure negligible water vapor concentration fluctuation in the optical path during spectral acquisition. As expected, the second derivative spectrum of 2,2,4-trimethylpentane in the 1800–1550 cm^−1^ spectral region is basically a flat line and sample’s absorbance-dependent water vapor interference is negligible.

The sample’s absorbance-dependent water vapor interference is a new phenomenon that has never been reported before. First, this phenomenon is not simply due to the deviation from Beer’s law of the measured absorbance of liquid H_2_O. A deviation from Beer’s law can result in the situation where water vapor interference can depend on sample’s absorbance as different regions of the sample’s absorption band deviate from Beer’s law to different extents. However, the absorption maxima of liquid H_2_O in Figure 1a is smaller than 1 and it is within the linear range of our FTIR spectrometer as evidenced in Figure S1. And even in the low-absorbing regions of liquid H_2_O such as the regions above 1700 cm^−1^ and below 1600 cm^−1^ in Figure 1a, we can still observe water vapor interference. Second, the sample’s absorbance-dependent water vapor interference is not simply due to the deviation from Beer’s law of the water vapor absorption. According to “resolution error theory” by Anderson and Griffiths [24,25], the absorption spectrum of water vapor measured under low spectral resolution (*i.e.*, 4 cm^−1^) does not follow Beer’s law. This means that the spectral subtraction between two different water vapor absorption spectra with one universal subtraction factor can never result in complete atmospheric compensation within the entire sample’s absorption band. However, in our study, we keep the concentration of water vapor constant with sample shuttle during spectral acquisition (as evidenced in Figure 2). Therefore, the “resolution error” issue is no longer an issue in our study.

To better understand this new phenomenon, we provide the following mathematical reasoning. As we know, the negative logarithm of the ratio of sample’s single-beam spectrum to reference’s single-beam spectrum gives an FTIR absorption spectrum. If we take the liquid H_2_O spectrum in Figure 1a as an example, the measured absorbance of liquid H_2_O taken under atmospheric conditions can in principle be expressed according to Equation (1). In Equation (1), *Abs*_*H*_2_*O*_(*measured*) is the measured value of liquid H_2_O’s absorbance; *Abs*_*H*_2_*O*_(*theoretical*) is the theoretical value of liquid H_2_O’s absorbance; the second term, log
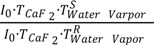
, is the water vapor interference term where *I*_0_ is the energy of infrared (IR) radiation from the IR source; *T*_*caF*_2__ is the transmittance of CaF_2_ window and is assumed to be identical for both sample scanning and reference scanning; 
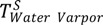
 is the transmittance of water vapor in sample scanning; 
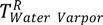
 is the transmittance of water vapor in reference scanning. If we assume a constant water vapor concentration level during spectral acquisition (this can be technically achieved as evidenced by the water-vapor-free 100% transmittance spectrum in Figure 2), 
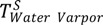
 and 
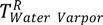
 will be equal and the log
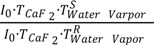
 term will be zero. In this way, the measured absorbance of liquid H_2_O, *Abs*_*H*_2_*O*_(*measured*), will be its true value, *Abs*_*H*_2_*O*_(*theoretical*). This situation is called complete atmospheric compensation and under such situation the measured absorption spectrum of liquid H_2_O will be free from water vapor interference. However, any spectral measurement is accompanied by noise. Therefore, in practice, the measured absorbance of liquid H_2_O, *Abs*_*H*_2_*O*_(*measured*), should be expressed according to Equation (2) by taking noise into account. In Equation (2), *Noise^s′^* is the noise level of the single-beam spectrum of sample scanning and *Noise^R^* is the noise level of the single-beam spectrum of reference scanning. If *Noise^s′^* and *Noise^R^* are equal, the log
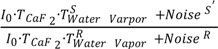
 term can still be zero at each frequency and the measured absorbance, *Abs*_*H*_2_*O*_(*measured*), will still be equal to its theoretical value of *Abs*_*H*_2_*O*_(*theoretical*). Yet, the real situation is that the layer of liquid H_2_O in the optical path actually behaves like an optical filter that attenuates the energy of IR radiation from the IR source. As spectral noise level depends on the energy of IR radiation [26], this will make *Noise^s′^* different from *Noise^R^*. Consequently, the water vapor interference term of log
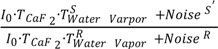
 is no longer zero and the measured absorbance of liquid H_2_O will contain the contributions from uncompensated water vapor absorptions even if 
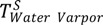
 = 
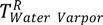
. Moreover, since the noise level varies with the absorbance of liquid H_2_O at each frequency, the water vapor interference term of log
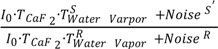
 will be different at each frequency within the absorption band. This will result in the so-called sample’s absorbance-dependent water vapor interference. The relationship of 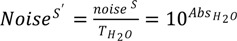 · *Noise^s^* also explains why the larger the absorbance of liquid H_2_O, the greater the water vapor interference. In this mathematical reasoning, we use noise as the origin of the additive term in Equation (2). Whether there is an alternative cause for the additive term is an open question and deserves future investigation.

We here take liquid H_2_O as an example for our reasoning, the sample’s absorbance-dependent water vapor interference phenomenon is apparently an issue inherent to any FTIR measurement including in the case of measuring protein FTIR spectrum whenever the measurement is taken under atmospheric conditions. By nature, the sample’s absorbance-dependent water vapor interference can be considered as a unique type of deviation from Beer’s law, but it is different from the deviation from Beer’s law in quantitative analysis in our conventional wisdom because the measured sample’s absorbance still follows Beer’s law. It is the second derivative spectrum that is significantly “deviated” from its true spectrum.

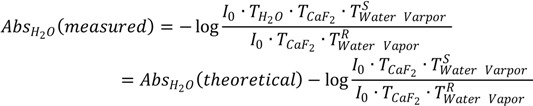
(1)

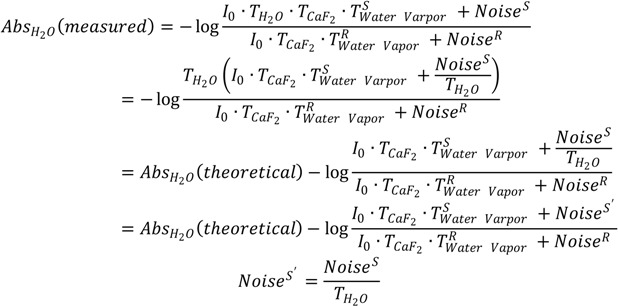
(2)


An immediate implication from the sample’s absorbance-dependent water vapor interference phenomenon is that the successful elimination of water vapor interference at several selected frequencies in the amide I region or from the 1850–1720 cm^−1^ window region cannot guarantee the successful elimination of water vapor interference from the entire amide I region because the extent of water vapor interference varies with protein’s absorbance at each frequency. Therefore, the above mathematical reasoning provides the theoretical basis for us to challenge the reliability of the established “single-point” criterion and “window-region” criterion.

**Figure 3:**
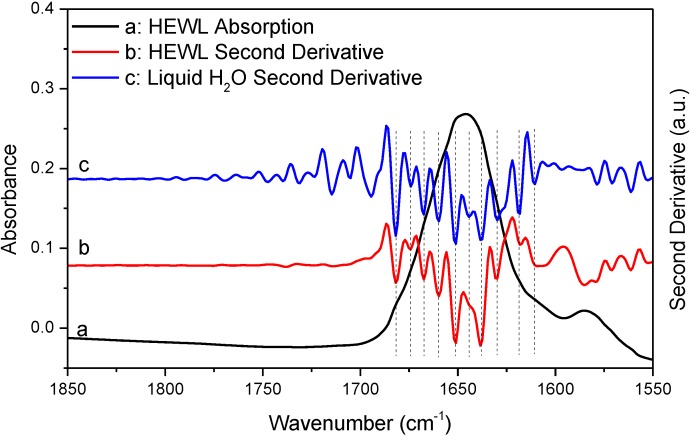
FTIR absorption (a) and second derivative (b) spectra of HEWL in D_2_O and second derivative spectrum of liquid H_2_O (c). Second derivative spectrum is displayed with its resolved peaks pointing downwards and is in arbitrary unit (a.u.). The negative *y*-axis offset observed in the HEWL absorption spectrum is due to a slight mismatch in thickness between sample IR cell and reference IR cell which results in imperfect solvent D_2_O subtraction. This has no effect on water vapor compensation between sample scanning and reference scanning [3]. Spectral resolution: 4 cm^−1^.

We now provide several examples to further question the reliability of the established “single-point” and “window-region” criteria. Figure 3 shows the absorption spectrum of deuterated hen egg white lysozyme (HEWL) amide I' band. The spectrum was obtained with sample shuttle to ensure negligible water vapor concentration fluctuation during spectral acquisition. HEWL is used here as a model protein. Though choosing HEWL is random, the nearly featureless amide I band of HEWL indeed makes HEWL a nice model protein when testing the effect of water vapor interference on the outcomes of second derivative and FSD. The absorption spectrum of HEWL apparently satisfies the established criteria for the elimination of water vapor interference. First, the original absorption spectrum looks rather smooth and contains no obvious features that can be assigned to water vapor absorption. Furthermore, none of the resolved peaks by second derivative technique matches the absorption peaks of water vapor in frequency as indicated in Table 1. These observations support that the HEWL spectrum satisfies the “single-point” criterion. Second, the 1850–1720 cm^−1^ window regions in both of the original spectrum and the second derivative spectrum in Figure 3 are featureless besides some spectral noises. This point is more explicitly illustrated in Figure S2 with the magnified window region. This means that the HEWL spectrum in Figure 3 also satisfies the “window-region” criterion. Based on the above observations, we can conclude that the HEWL spectrum in Figure 3 satisfies all of the established criteria and should be considered as being negligibly affected by water vapor interference. Consequently, the resolved peaks in the second derivative spectrum of HEWL should be assigned to protein secondary structures. Yet, the comparison between the two second derivative spectra of HEWL and liquid H_2_O in Figure 3 obviously contradicts such conclusion. As indicated by the vertical lines in Figure 3, all the peaks resolved by second derivative technique from HEWL spectrum match the resolved peaks (*i.e.*, the artifact peaks due to water vapor interference) from liquid H_2_O very well. In Table 1, we list the two sets of frequencies to further illustrate this point. This observation serves as strong evidence to support that a protein absorption spectrum judged by the established criteria to be water vapor interference free can still be significantly affected by water vapor interference. In Table 1, one may wonder why the actual frequencies of the resolved peaks due to water vapor interference do not match the frequencies of water vapor absorption bands. This is due to the fact that the resolved peak frequencies and even the number of the resolved peaks by second derivative technique highly depend on window size in Savitzky-Golay algorithm used for second derivative treatment and zero-filling factor. Realizing this point is critical. Otherwise, we may falsely conclude that the resolved peaks in the second derivative spectrum of HEWL are the component peaks due to protein secondary structures because these peaks do not match the absorption peaks of water vapor in frequency according to the “single-point” criterion.

**Table 1:** Frequencies of the resolved peaks in the second derivative spectra of HEWL and liquid H_2_O and frequencies of the absorption peaks of water vapor in the amide I region.

Spectrum	Peak Frequency (cm^−1^)							
HEWL (second derivative)	1682	1674	1667	1660	1651	1638	1630	1618
Liquid H_2_O (second derivative)	1682	1674	1667	1660	1651	1638	1630	1618
Water Vapor (absorption)	1684	1670	1663	1653	1647	1636	1623	1616

**Figure 4:**
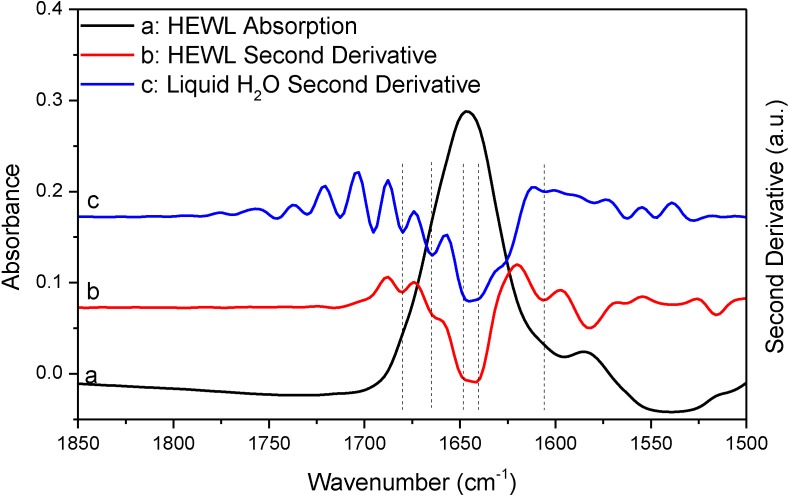
FTIR absorption (a) and second derivative (b) spectra of HEWL in D_2_O and second derivative spectrum of liquid H_2_O (c). Second derivative spectrum is displayed with its resolved peaks pointing downwards and is in arbitrary unit (a.u.). Spectral resolution: 8 cm^−1^.

**Figure 5:**
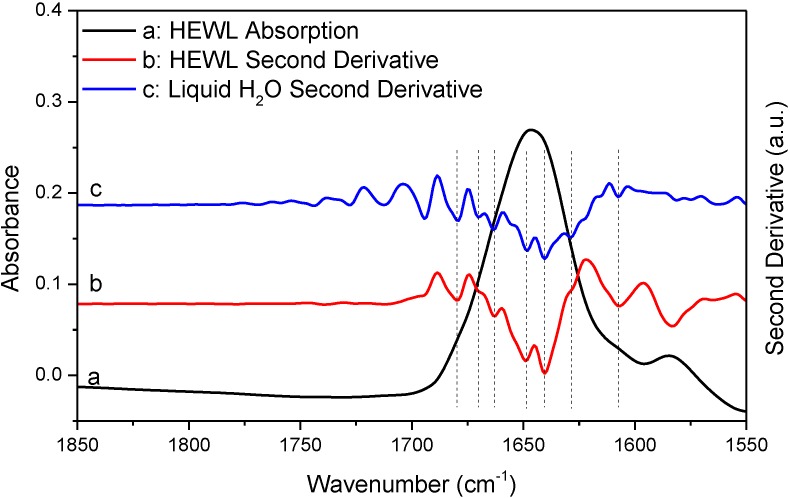
FTIR absorption (a) and second derivative (b) spectra of HEWL in D_2_O and second derivative spectrum of liquid H_2_O (c). Second derivative spectrum is displayed with its resolved peaks pointing downwards and is in arbitrary unit (a.u.). Both of the spectra of HEWL and liquid H_2_O were subjected to 17-point smoothing. Spectral resolution: 4 cm^−1^.

In Figure 4 and Figure 5, we performed additional comparative studies of HEWL and liquid H_2_O under different conditions, *i.e.*, using 8 cm^−1^ spectral resolution to collect the absorption spectrum of HEWL and using smoothing to pretreat the absorption spectrum of HEWL before second derivative treatment. Figure 4 shows the HEWL absorption spectrum collected with 8 cm^−1^ resolution and its second derivative; Figure 5 shows the HEWL absorption spectrum after 17-point smoothing and its second derivative. In Figure 4 and Figure 5, the second derivative spectra of liquid H_2_O that are subjected to the same acquisition condition and spectral treatment are shown for comparison. The two HEWL absorption spectra apparently satisfy both the “single-point” criterion and the “window-region” criterion. This is as expected because low spectral resolution and smoothing are known to be able to further suppress water vapor interference. Yet, the perfect match in frequency between the second derivative spectra of HEWL and liquid H_2_O as illustrated by the vertical lines in Figure 4 and Figure 5 again supports that a protein spectrum satisfying the established criteria is still affected by water vapor interference significantly. It also indicates that the perfect match in frequency between the two sets of resolved peaks of HEWL and liquid H_2_O in Figure 3 is not just a coincidence. Besides the HEWL system, we also investigated two additional protein systems, bovine serum albumin and cytochrome c. The two absorption spectra shown in Figures S3 and S4 satisfy both the “single-point” criterion and the “window-region” criterion. Yet, the two spectra are still significantly affected by water vapor interference as evidenced through the comparison between the second derivative spectra of protein and liquid H_2_O. These observations again support our argument that the established criteria cannot offer reliable evaluation of water vapor interference in the protein amide I band.

**Figure 6:**
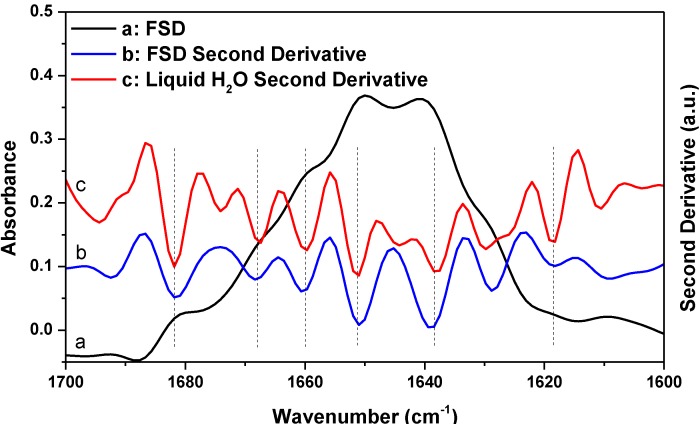
FSD spectrum of HEWL in D_2_O (a) and its corresponding second derivative (b) and second derivative spectrum of liquid H_2_O (c). Second derivative spectrum is displayed with its resolved peaks pointing downwards and is in arbitrary unit (a.u.). Spectral resolution: 4 cm^−1^.

Besides second derivative, FSD is another widely used resolution-enhancement technique in protein secondary structural analysis. To see whether the FSD result also supports the argument that a protein spectrum satisfying the established criteria for successful water vapor absorption elimination can still be significantly affected by water vapor interference, we performed a comparative FSD study of HEWL and liquid H_2_O, as shown in Figure 6. The FSD treatment was performed on the HEWL absorption spectrum shown in Figure 3 using Lorentzian bandwidth of 20 cm^−1^ and noise suppression factor of 0.3. FSD is a subjective technique and there is no consensus in the literature on what should be the most appropriate values for bandwidth and noise suppression factor in FSD for protein secondary structure analysis. The two parameters for Figure 6 had been used previously for protein spectra taken with Bruker’s FTIR spectrometer [27]. In Figure 6, we further performed second derivative treatment on the FSD spectrum as the exact positions of the component peaks in the FSD spectrum often need to be determined by further second derivative treatment on the FSD spectrum [27]. As indicated by the vertical lines in Figure 6, the peaks resolved with FSD from HEWL match the resolved artifact peaks due to water vapor interference from liquid H_2_O very well. Therefore, the FSD study also supports our argument that the previously established criteria for successful elimination of water vapor interference are not reliable.

Since the above mathematical reasoning demonstrates that both the “single-point” and “window-region” criteria are problematic, it is thus desirable to develop some type of “whole-spectrum” criterion to better evaluate the extent of water vapor interference. In fact, the comparative second derivative study of protein and liquid H_2_O has provided us a solution. We can employ such comparison as a new criterion to more reliably evaluate water vapor interference. This new criterion can be implemented in the following way. First, we take the absorption spectra of a protein sample and liquid H_2_O under identical acquisition conditions and then make them subject to the same data processing such as smoothing and spectral subtraction. Second, we perform a comparison between the second derivative spectra of protein and liquid H_2_O. There are two possible situations that one would face. If we do have a perfect elimination of water vapor interference, the second derivative spectrum of liquid H_2_O will be like that of liquid D_2_O, showing only one component peak. Then under this situation, the resolved peaks from the second derivative spectrum of protein should be identified to be the true component peaks due to protein secondary structures. If we do not have a perfect elimination of water vapor interference, we will search for the matched peaks between the second derivative spectra of protein and liquid H_2_O like what we have done in Figure 3, Figure 4 and Figure 5. The resolved peak in the second derivative spectrum of protein that matches the artifact peak in the second derivative spectrum of liquid H_2_O in frequency should be considered as artifacts due to water vapor interference (or at least suspected as coincidence does exist occasionally). Besides frequency matching, the similarity in shape between the two matched peaks is another piece of strong evidence for us to confirm the identity of the artifact peak due to water vapor interference.

Before we conclude, there are two additional issues that we would like to address particularly with respect to the implications of our work for the FTIR analysis of protein secondary structures. First, we want to emphasize that though we proposed a new criterion in this study and also questioned the reliability of the established criteria, we have no intent to say that the previously established criteria should be abandoned. In practice, the previously established criteria and the newly proposed criterion can work together to ensure a more reliable second derivative or FSD treatment on protein absorption spectrum. Second, we would like to state that water vapor interference needs not to be a serious concern in every FTIR analysis of protein secondary structure. As we know, there are three types of FTIR approaches in protein secondary structural analysis. The first one is the FTIR curve-fitting approach with the aid of second derivative and FSD. With this approach, the initial guess about the frequencies and the number of the component peaks are based on the results from second derivative or FSD treatment. The actual curve-fitting can be performed on the original spectrum, the inverted second derivative spectrum, or the FSD spectrum. This type of approach is the one that we have been discussing in this study and it is the widely used one compared to the latter two. The second approach is through the curve-fitting of the original absorption spectrum without the aid of second derivative or FSD [28,29,30]. This is a direct-fit approach. With this approach, the initial guess about the frequencies and the number of the component peaks are not based on the results from second derivative or FSD treatment. The third approach is the chemometric approach based on multivariate statistical analysis [31,32,33,34,35,36]. Both of the direct-fit approach and the chemometric approach can be immune to water vapor interference as long as the absorption spectrum is in decent quality without obvious spikes from water vapor. Furthermore, for the chemometric approach, as it is a pure mathematical treatment, it is immune to water vapor interference even though the second derivative or FSD spectra are used in the statistical analysis. Therefore, for the latter two approaches, the previously established criteria are good enough to ensure the FTIR analysis is negligibly affected by water vapor interference. By contrast, for the first FTIR approach involving second derivative or FSD treatment, we do need to have more rigorous criteria to evaluate the effect of water vapor interference. Otherwise, the resolved peaks by second derivative or FSD techniques may largely be the artifacts due to water vapor interference as we have illustrated in Figure 3, Figure 4, Figure 5 and Figure 6. In this respect, the direct-fit approach and the chemometric approach are advantageous over the second derivative-based or FSD-based curve-fitting approach in protein secondary structural analysis.

## 3. Experimental Section

### 3.1. Materials

Hen egg white lysozyme (catalog number, L6876) was obtained from Sigma-Aldrich (St. Louis, MO, USA). Deionized water with a resistivity of 18.2 MΩ·cm was obtained from a Millipore system (Billerica, MA, USA). Deuterium oxide (D_2_O) with a purity of >99.8% was obtained from J&K Chemical (Beijing, China). 2,2,4-Trimethylpentane with a purity of 99% was obtained from Sigma-Aldrich.

### 3.2. Sample Preparation

The FTIR spectrum of HEWL in solution was measured in D_2_O. Deuteration of HEWL was performed according to a literature protocol [37]. Briefly, the solution containing 20 mg/mL HEWL is incubated at 70 °C in a thermo-shaker for 15 min and then rapidly cooled to room temperature. The disappearance of the amide II band of lysozyme at 1540 cm^−1^ confirms full H/D exchange.

### 3.3. FTIR Measurement

The FTIR spectra were obtained with a Bruker Vertex 70 FTIR spectrometer (Bruker, Karlsruhe, Germany). The spectrometer is equipped with a DTGS detector. The interferometer is a cube corner design. A Bruker-made sample shuttle (Model: A508/Q) is installed inside the sample compartment. The sample shuttle is an FTIR sampling accessory, which can provide interleaved sample and reference single-beam scanning in transmission mode without the need of opening sample compartment for sample change which could cause atmospheric perturbation. Sample shuttle can ensure constant water vapor concentration level during spectral acquisition thus result in complete atmospheric compensation. As shown in Figure 2, the water-vapor-free 100% transmittance line taken under our lab conditions can be repeatedly obtained with ease, demonstrating the efficacy of sample shuttle in this study.

To obtain a FTIR spectrum of a liquid sample, demountable CaF_2_ liquid cells are used. In particular, to obtain the absorption spectrum of HEWL in D_2_O, the sample cell contains protein solution in D_2_O and the reference cell contains solvent D_2_O. Fifty micrometers spacer is used for both sample cell and reference cell. To obtain the absorption spectrum of liquid H_2_O or D_2_O, the sample cell contains H_2_O or D_2_O and the reference cell is empty cell. No spacer is used due to the strong absorption of water bending mode. To obtain the absorption spectrum of 2,2,4-trimethylpentane, the sample cell contains 2,2,4-trimethylpentane and the reference cell is empty. A 50 µm spacer is used for sample cell. The absorption spectrum of water vapor was obtained by introducing some atmospheric fluctuation between sample scanning and reference scanning of the empty optical path.

Spectral processing such as second derivative and FSD was performed using Bruker’s OPUS software (Version 7.2). The default window size in Savitzky-Golay algorithm for second derivative treatment is 9-point in OPUS software. Savitzky-Golay algorithm is also used for smoothing. All FTIR measurements were performed under ambient conditions. Typical acquisition parameters are listed below: spectral resolution, 4 cm^−1^; scan number, 32; zero-filling factor, 4; apodization function, Blackman-Harris 3-Term; phase resolution, 16; phase correction mode, Mertz; aperture, 6 mm; scan speed, 10 kHz; acquisition mode, double-sided, forward-backward. The FTIR spectra presented here were all taken under the above acquisition condition unless otherwise mentioned. Besides the typical condition, we have run our experiments under other acquisition conditions such as using different scan number (*i.e.*, 128) (Figure S5), using different zero-filling factor (*i.e.*, 2) (Figure S6), using MCT detector (Figure S7), using different apodization function (Figure S8), using a different aperture (*i.e.*, 3 mm) (Figure S9), and using a different FTIR spectrometer (*i.e.*, Thermo-Nicolet 6700 with a dynamically aligned interferometer design) (Figure S10). The results shown in Figure S5 to Figure S10 support that the conclusion in this study is independent of the actual condition that we chose.

## 4. Conclusions

In this study, we challenged the reliability of the established criteria for the evaluation of water vapor interference in protein secondary structural analysis by FTIR spectroscopy through a comparative study of protein and liquid water. We explained why the established criteria are not reliable using mathematical reasoning and introduced a new concept called sample’s absorbance-dependent water vapor interference to support our argument. We then proposed a new criterion based on the comparison between the second derivative spectra of protein and liquid water to better evaluate water vapor interference. We suggest that people use this newly proposed criterion to evaluate the extent of water vapor interference in their FTIR spectra to ensure more reliable second derivative or FSD treatment on the protein amide I band during FTIR curve-fitting analysis.

## Supplementary Files

Supplementary File 1 Supplementary Information (PDF, 220 KB)
