# Supplementary material for: A New Criterion to Evaluate Water Vapor Interference in Protein Secondary Structural Analysis by FTIR Spectroscopy
Source: Int J Mol Sci. 2014 Jun 4;15(6):10018–33. doi: 10.3390/ijms150610018 (PMC4100137; doi:10.3390/ijms150610018)
Supplement: Supplementary File 1 — Supplementary Information (PDF, 220 KB) [file ijms-15-10018-s001.pdf]

Supplementary Information

Figure S1. (**a**) FTIR absorption spectra of water in DMSO in the H_2O bending mode region measured with IR cells in five different thicknesses; (**b**) The linear relationship between absorbance and thickness. The absorbance at maxima is used for the linear fitting. Figure S1 is to determine the linear range of our FTIR spectrometer according to Beer's law. Water in DMSO is used for this purpose. The FTIR spectra of H_2O bending mode in the 1800–1500 cm^{-1} region were measured using IR cells in five different thicknesses. This experiment shows that even with an absorbance value of ~1.1, the Beer's law is still satisfied.

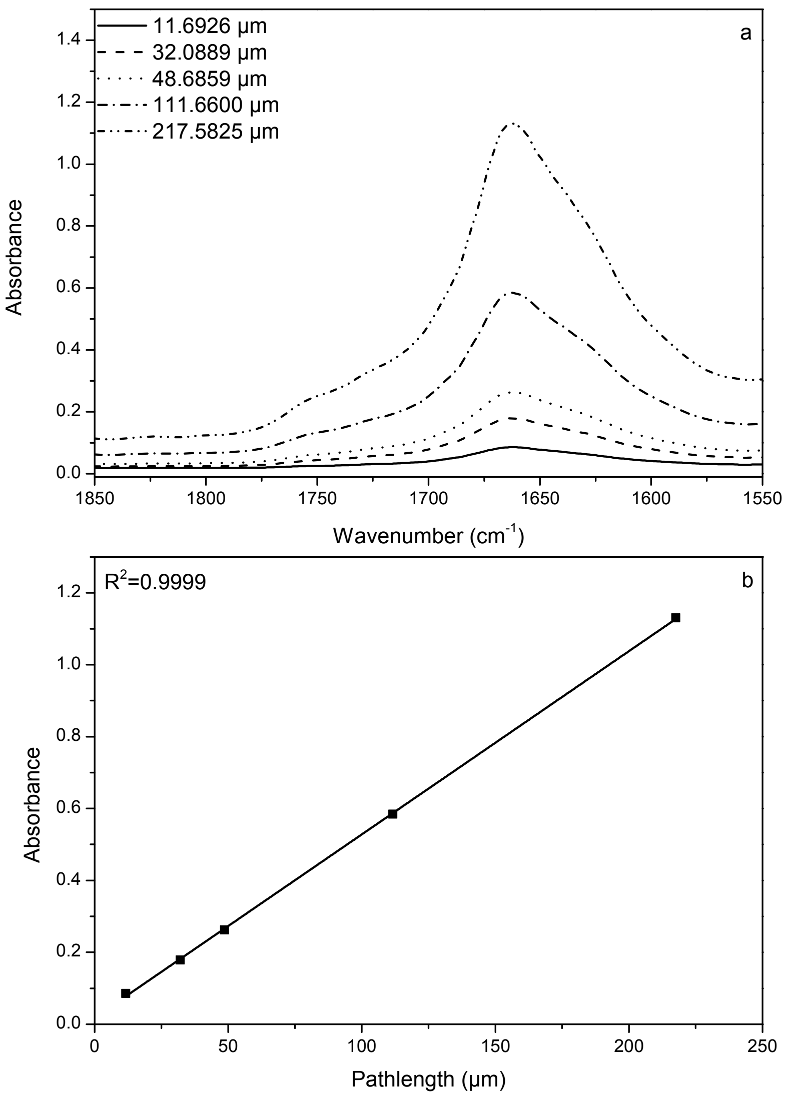

Figure S2. FTIR absorption (black curve) and second derivative (red curve) spectra of HEWL and absorption spectrum of water vapor (blue curve) in the 1850–1720 cm^{-1} spectral region. The *y*-axis is significantly magnified. The peaks in the second derivative spectrum are denoted with asterisks. Vertical lines show the positions of the water vapor absorptions. This figure is part of Figure 3 in the manuscript.

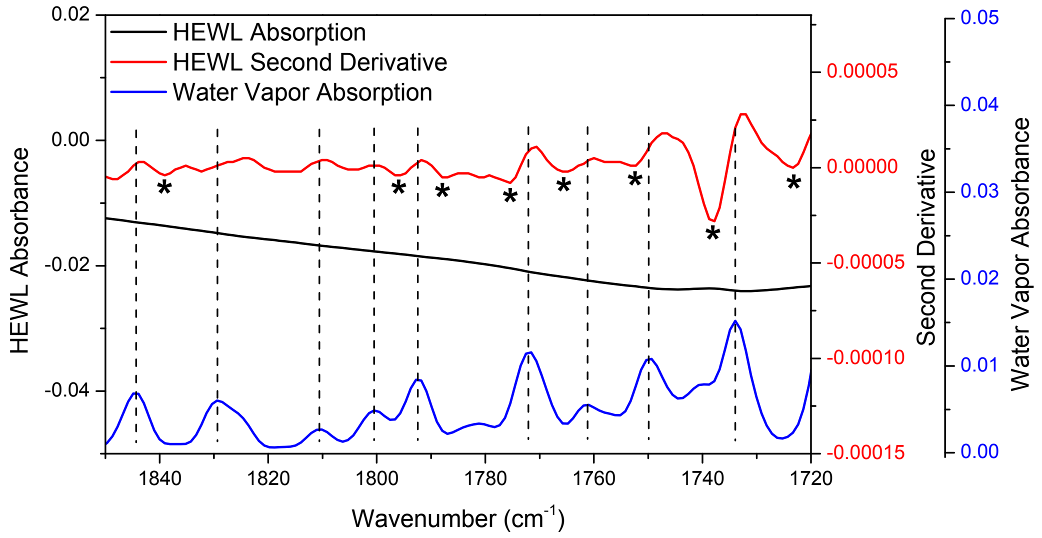

Figure S3. FTIR absorption (a) and second derivative (b) spectra of bovine serum albumin (BSA) in D_2O and second derivative spectrum of liquid H_2O (c). Second derivative spectrum is displayed with its resolved peaks pointing downwards. Acquisition parameters and data manipulations are the same as that in Figure 3 for HEWL. BSA was obtained from Roche (Switzerland). Preparation of deuterated BSA solution in D_2O was performed in the same manner as that for HEWL. There are some unchanged hydrogens after H/D exchange due to the relatively stable structure of BSA, but this has no effect on the evaluation of the established criteria. Protein concentration of BSA was determined to be 20 mg/mL by weighing. The vertical lines show the perfect match in frequency between the resolved peaks in the two second derivative spectra.

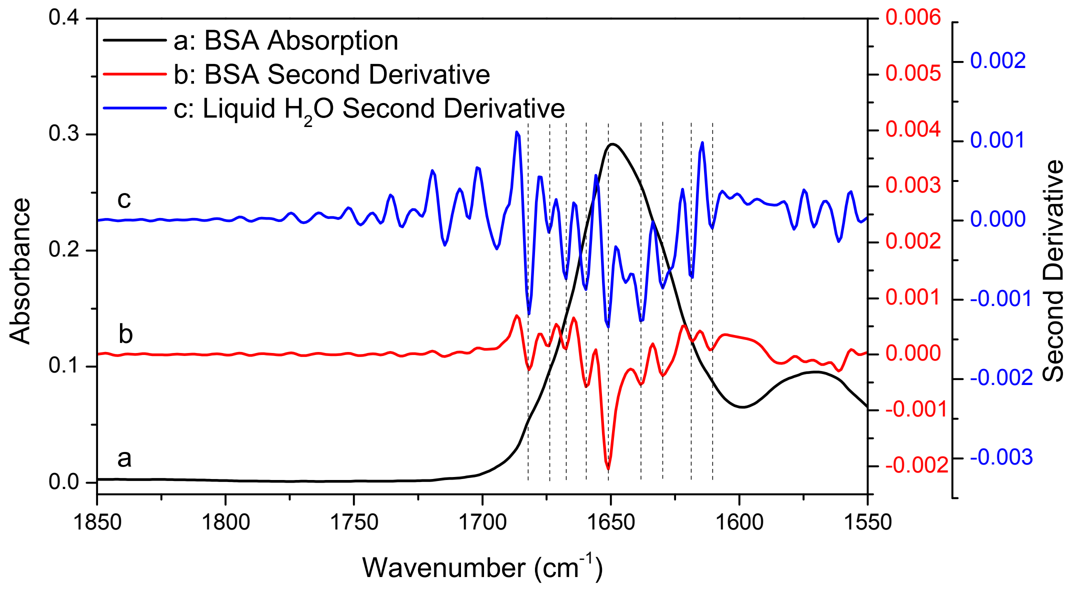

Figure S4. FTIR absorption (a) and second derivative (b) spectra of cytochrome c in D_2O and second derivative spectrum of liquid H_2O (c). Second derivative spectrum is displayed with its resolved peaks pointing downwards. Acquisition parameters and data manipulations are the same as that in Figure 3 for HEWL. Cytochrome c was obtained from Sigma-Aldrich (Saint Louis, MO, USA). Preparation of deuterated cytochrome c solution in D_2O was performed in the same manner as that for HEWL. There are some unchanged hydrogens after H/D exchange due to the relatively stable structure of cytochrome c, but this has no effect on the evaluation of the established criteria. Protein concentration of cytochrome c was determined to be 20 mg/mL by weighing. The vertical lines show the perfect match in frequency between the resolved peaks in the two second derivative spectra.

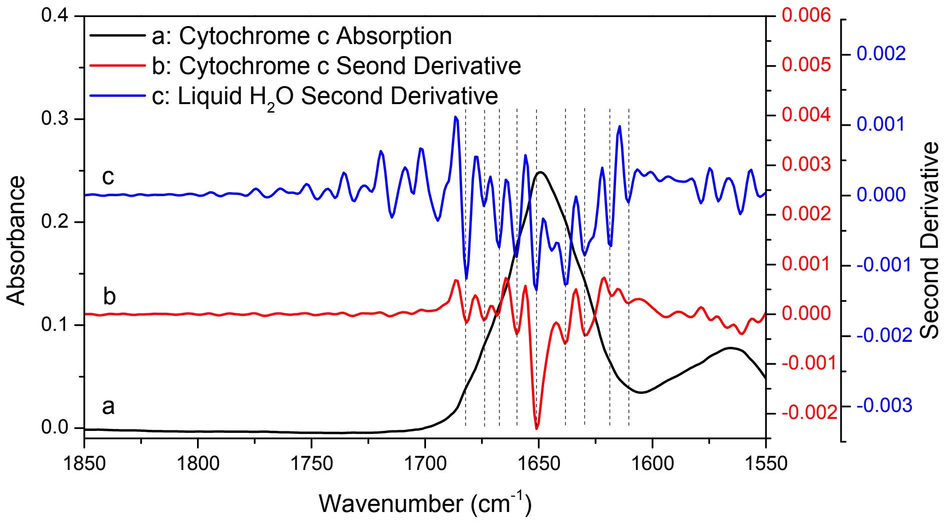

Figure S5. FTIR absorption (a) and second derivative (b) spectra of HEWL and second derivative spectrum of liquid H_2O (c) in the 1850–1550 cm^{-1} spectral region. Acquisition parameters are the same as that in Figure 3 in the manuscript except that the scan number is 128. The vertical lines show the perfect match in frequency between the resolved peaks in the two second derivative spectra.

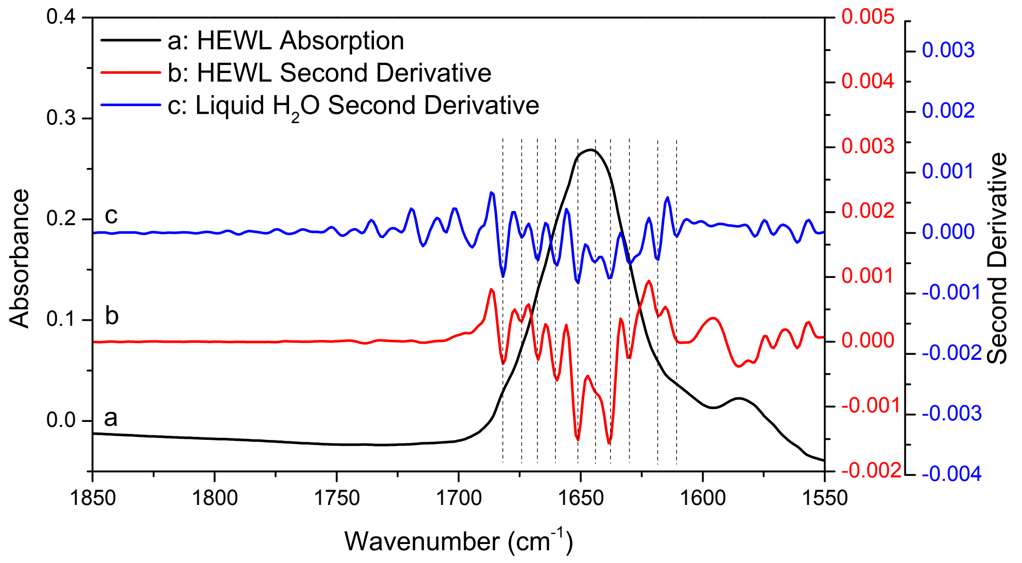

Figure S6. FTIR absorption (a) and second derivative (b) spectra of HEWL and second derivative spectrum of liquid H_2O (c) in the 1850–1550 cm^{-1} spectral region. Acquisition parameters are the same as that in Figure 3 in the manuscript except that the zero-filling factor is 2. The vertical lines show the perfect match in frequency between the resolved peaks in the two second derivative spectra.

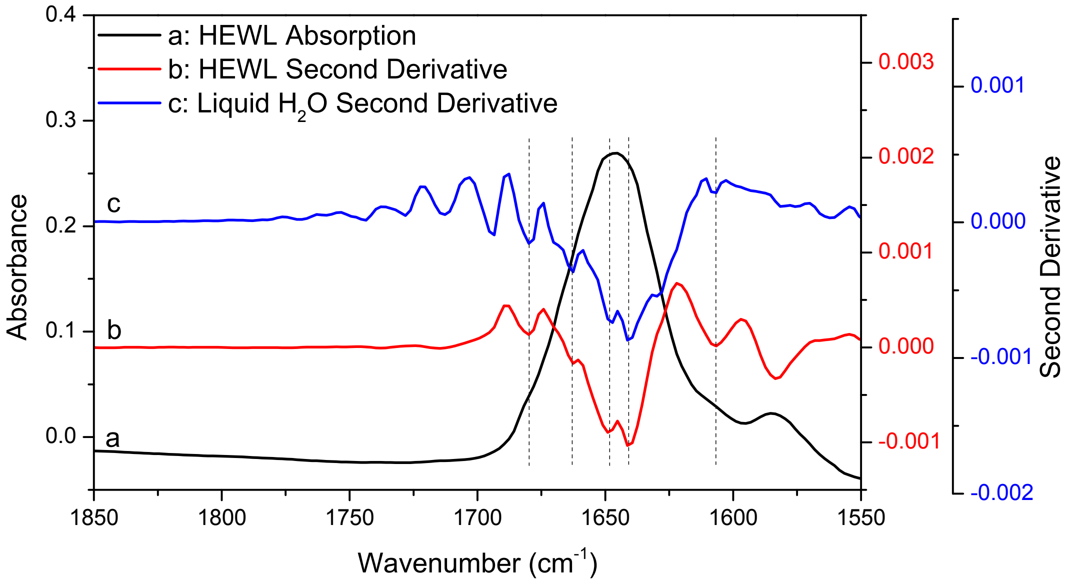

Figure S7. FTIR absorption (a) and second derivative (b) spectra of HEWL and second derivative spectrum of liquid H_2O (c) in the 1850–1550 cm^{-1} spectral region. Acquisition parameters are the same as that in Figure 3 in the manuscript except that a MCT detector is used. The default and ideal aperture setting recommended by Bruker is 1 mm for MCT detector. An IR cell with a 25 µm spacer was used for the measurement of HEWL absorption spectrum. The vertical lines show the perfect match in frequency between the resolved peaks in the two second derivative spectra.

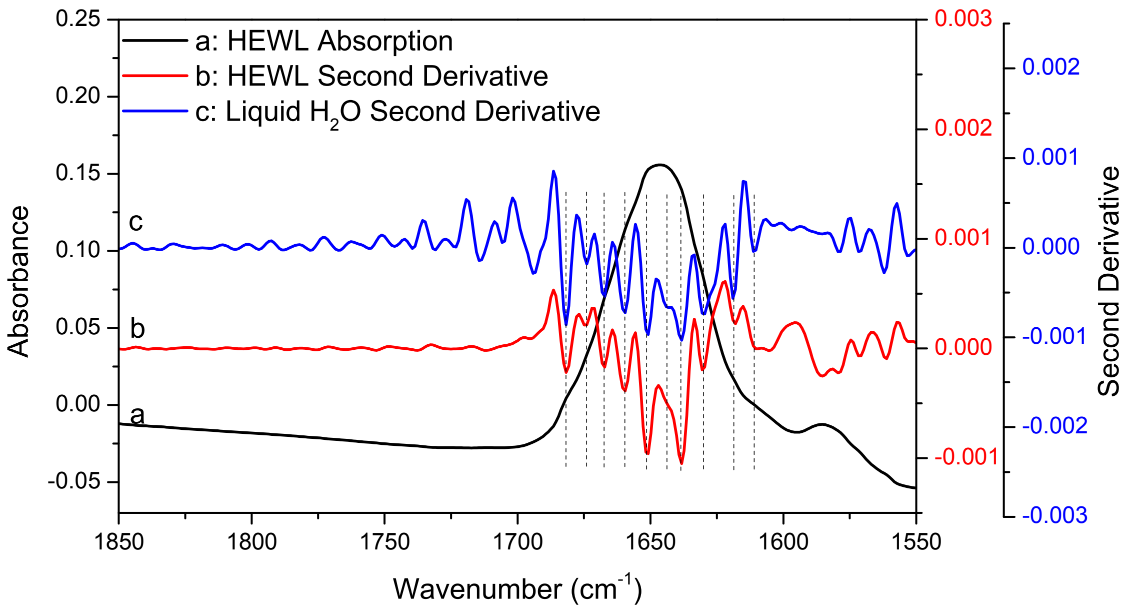

Figure S8. FTIR absorption (a) and second derivative (b) spectra of HEWL and second derivative spectrum of liquid H_2O (c) in the 1850–1550 cm^{-1} spectral region. (**A**) using Happ-Genzel function; (**B**) using Norton-Beer Medium function. Acquisition parameters are the same as that in Figure 3 in the manuscript except that a different apodization function is used. The vertical lines show the perfect match in frequency between the resolved peaks in the two second derivative spectra.

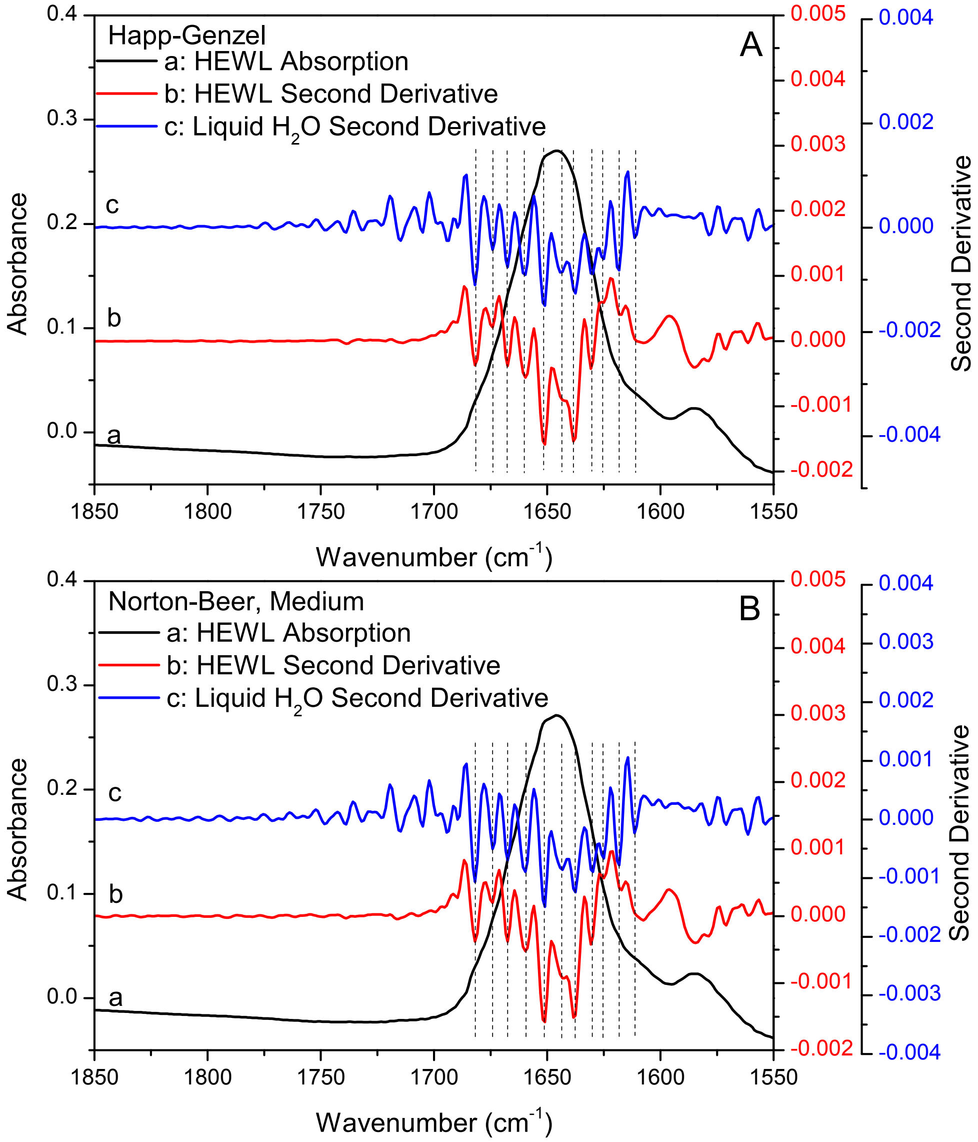

Figure S9. FTIR absorption (a) and second derivative (b) spectra of HEWL and second derivative spectrum of liquid H_2O (c) in the 1850–1550 cm^{-1} spectral region. Acquisition parameters are the same as that in Figure 3 in the manuscript except that a 3 mm aperture is used. The default and ideal aperture setting recommended by Bruker is 6 mm for DTGS detector. The vertical lines show the perfect match in frequency between the resolved peaks in the two second derivative spectra.

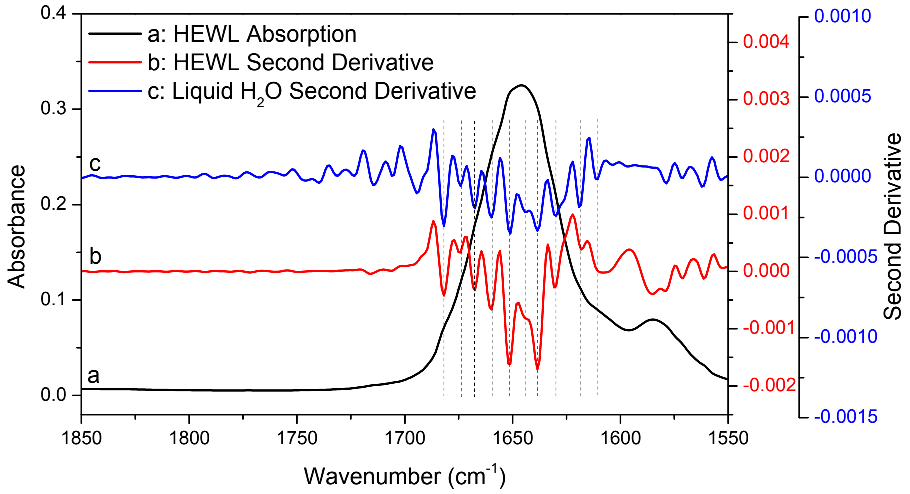

Figure S10. FTIR absorption (a) and second derivative (b) spectra of HEWL and second derivative spectrum of liquid H_2O (c) in the 1850–1550 cm^{-1} spectral region. A Thermo-Nicolet 6700 FTIR spectrometer is used. Acquisition parameters are as follows: detector: MCT; spectral resolution: 4 cm^{-1}; scan number: 32; zero-filling factor: 1-Level; apodization function: Blackman-Harris; sample shuttle: installed. The second derivative treatments were performed with Nicolet's Omnic software using a 9-point window and a 5-order polynomial function in Savitzky-Golay algorithm. The vertical lines show the perfect match in frequency between the resolved peaks in the two second derivative spectra.

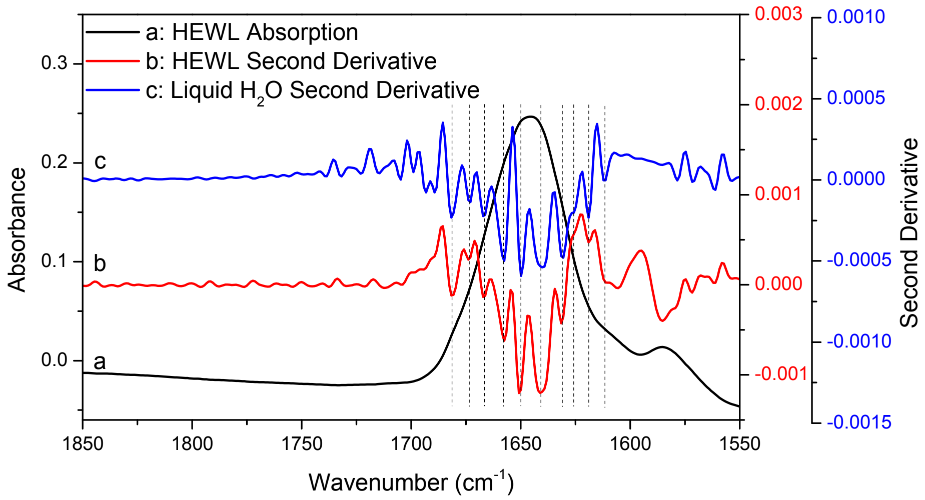

© 2014 by the authors; licensee MDPI, Basel, Switzerland. This article is an open access article distributed under the terms and conditions of the Creative Commons Attribution license (http://creativecommons.org/licenses/by/3.0/).
